# Report of a fatal case of Lassa fever in Parakou in 2018: clinical, therapeutic and diagnostic aspects

**DOI:** 10.1186/s12879-018-3587-6

**Published:** 2018-12-17

**Authors:** Cossi Angelo Attinsounon, Bienvenu Rolland Ossibi Ibara, Adébayo Alassani, Serge Adé, Khadidjatou Saké, Clément Glèlè Kakaï, Albert Dovonou

**Affiliations:** 1 Teaching and Research Unit of Infectious and Tropical Diseases, Faculty of Medicine, University of Parakou, 03 P. O Box 112, Parakou, Republic of Benin; 2 Laboratory of Expertise and Research in Tropical Infectiology, University of Parakou, 03 P. O Box 112, Parakou, Republic of Benin; 3 Medicine and Medical Specialities Department, Faculty of Medicine, University of Parakou, P. O Box 123, Parakou, Republic of Benin; 4 Public Health’s Department, Ministry of Health, P. O Box 01-882, Cotonou, Republic of Benin

**Keywords:** Lassa fever, Viral hemorrhagic fever, Clinical signs, Treatment, Diagnosis, Parakou, Benin

## Abstract

**Background:**

Lassa fever is one of the most lethal neglected tropical diseases in West Africa. It is a serious public health problem in this region of Africa where it is endemic in several countries. However, it remains a very little known disease by healthcare workers. The lack of specificity of its clinical manifestations makes its diagnosis difficult even in an epidemic context.

**Case presentation:**

We report here a confirmed case of Lassa fever whose diagnosis could not be suspected until 11 days after the symptomatology began. This case was recognized as a suspected case of Lassa fever in the Internal Medicine Department of the Regional and Teaching Hospital of Borgou due to the persistence of the fever and the worsening of the patient’s clinical condition despite triple antibiotic therapy in general and especially due to the appearance of hemorrhages. Confirmation of the presence of Lassa fever virus by Reverse Transcriptase Polymerase Chain Reaction (RT-PCR) assay on blood sample was obtained after his death despite late initiation of Ribavirin treatment.

**Conclusion:**

This case challenges Benin’s health authorities on the need to facilitate access to diagnosis of viral hemorrhagic fevers and to train caregivers at all levels of the health system for better management of these diseases.

## Background

Discovered in 1969 in a Nigerian nurse from the town of Lassa in the state of Borno, the arenavirus named Lassa virus is one of the viruses responsible for deadly epidemics of hemorrhagic fever in Africa [1].

Since the first epidemic was discovered in 2014 in the department of Atacora, Benin experienced its fourth epidemic of Lassa hemorrhagic fever in January 2018, with an average lethality of about 45% [2, 3].

We describe here the clinical presentation and therapeutic history of a confirmed case of Lassa fever diagnosed late in the internal medicine department of the Regional and Teaching Hospital of Borgou.

The aim of this study is to draw the attention of the healthcare workers to the clinical manifestations that a patient with Lassa fever may present so that they are more vigilant not only during epidemic periods but also outside them.

## Case presentation

This was a 34-year-old man, grower and farmer residing in the commune of Tchaourou. A Christian, he is married to two women and has seven children. He was hospitalized on 18 January 2018 with bloody and febrile diarrhea.

The beginning of symptomatology would date back to 10 January 2018 (approximately 1 week before admission) and was marked by the start of diarrhea, made up of soft, yellowish, sometimes mucusy, blood-ribbed stools, emitted three or four times a day, associated with a constant unquantified fever. The patient consulted on January 12, 2018 at his local health center where he received outpatient treatment consisting of: paracetamol 1 g × 2 per day, quinine 300 mg × 3 / day, metronidazole 500 mg × 2 / day, ciprofloxacin 500 mg × 2 / day, diazepam 5 mg in the evening, albendazole 400 mg in single dose. No paraclinical investigation has been conducted at this stage. Under this treatment, the course was marked by an improvement in symptoms for about 2 days but with the persistence of an unquantified low grade fever. Faced with the resumption on 16 January 2018 of the initial symptomatology and the appearance of two episodes of bilious vomiting, odynophagia, intense headaches and generalized aches, the patient was taken by his parents on 18 January 2018 to the Emergency Department of the Regional and Teaching Hospital of Borgou which referred him to the Internal Medicine Department for further management.

At admission, the interview did not note any particular medical history. The patient was non-smoker and would occasionally take alcohol.

Initial clinical examination revealed a temperature of 39 °C, tachycardia at 86 beats per minute, polypnea at 30 cycles per minute and blood pressure at 130 / 100 mmHg. Palpebral mucosas were well colored. There was no jaundice or edema of the lower limbs. Examination of the digestive tract revealed inflammation of the palatal tonsils covered with a whitish coating and pain in the right iliac fossa, which was not very intense and without radiation. The rectal exam noted a tonic anal sphincter and the finger came back with mucus. The cardiovascular examination noted a deafening of the heart sounds. Otherwise, the physical examination did not reveal any anomalies. The paraclinical check-up carried out at admission was as follows: a thick drop for trophozoites of negative plasmodium, red blood cells at 4.53 10^6^ / mm^3^, hemoglobin at 15.33 g/dL, hematocrit at 41.6%, platelets at 123 10^9^/L, leukocytes at 6.6 10^9^ cells/L with neutrophils at 70%, lymphocytes at 27%, eosinophils at 1% and monocytes at 2%, azotemia at 0.65 /L, creatininemia at 14.13 mg/L, natremia at 135.7 meq/L, kalemia at 4.08 meq/L, blood chloride at 115.4 meq/L, alanine aminotransferases (ALT) at 82 IU/L, aspartate aminotransferases (AST) at 62 IU/L. HIV status was negative.

In front of this clinical presentation, the diagnosis of severe sepsis was retained and the treatment instituted was ceftriaxone 2 g daily, metronidazole, intravenous (IV), 500 mg two times daily, gentamycin 3 mg/kg once daily, tramadol injection 100 mg two times daily, serum glucose 5%, saline 9‰ and oxygen therapy.

**Fig. 1:**
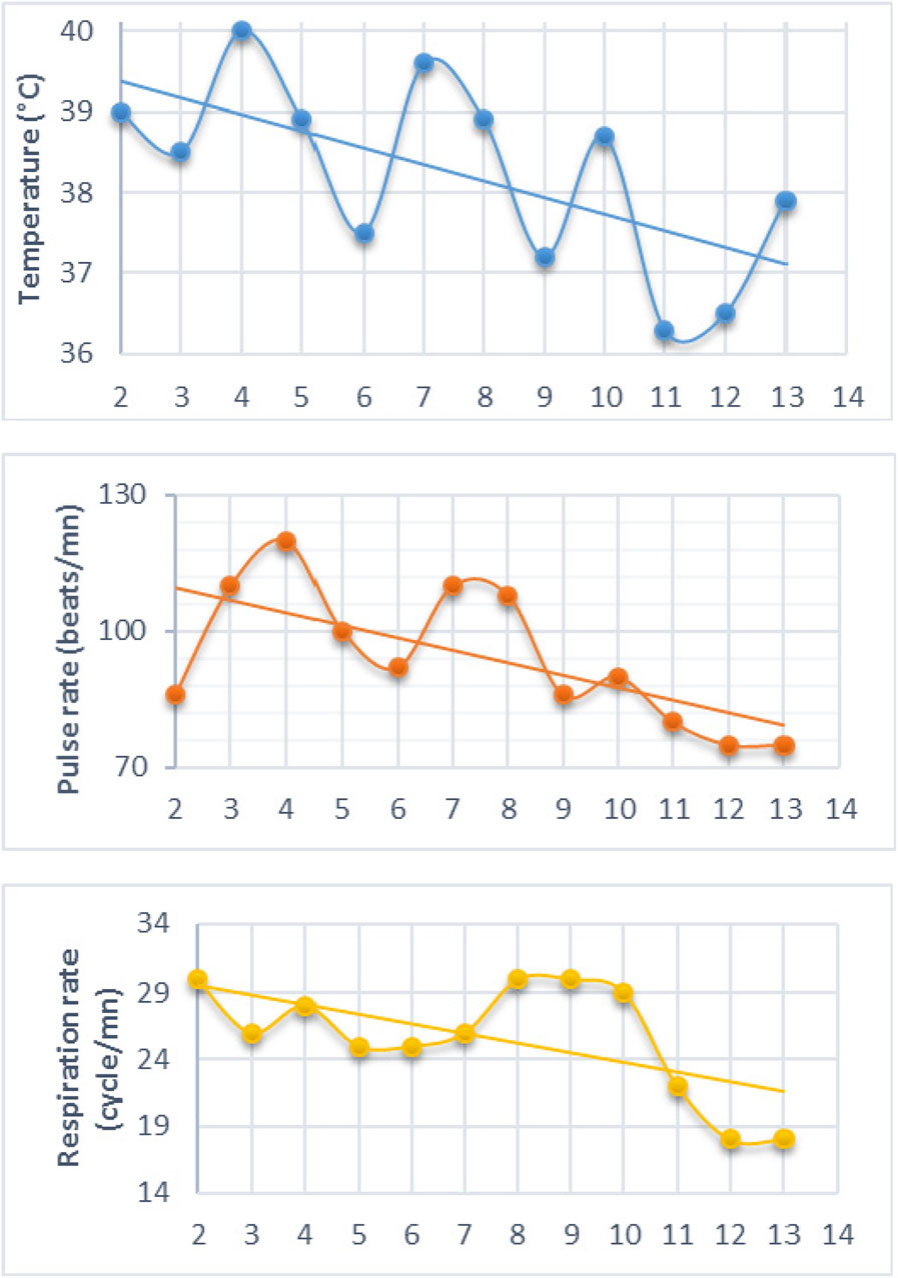
Vital signs (body temperature, pulse rate and respiration rate) of the patient during hospitalization in the Internal Medicine Department at the Regional and Teaching Hospital of Borgou

The course of this treatment was marked by persistent signs with fever ranging from 36.3 °C to 40.1 °C. Figure 1 shows core temperature (°C), pulse and respiratory rates (per minute) measured every 12 h throughout the hospitalization period for the patient. The occurrence on the third day of hospitalization of melena, facial puffiness and conjunctival hemorrhage gave rise to suspicion of viral hemorrhagic fever. Then, an insistent interrogation noted a regular consumption of rat. The patient was isolated and a blood sample was taken and sent to the national laboratory of viral hemorrhagic fever on 22 January 2018. He was given ribavirin 30 mg/kg IV (maximum, 2 g) loading dose to be followed by 15 mg/kg IV (maximum, 1 g) every 6 h for 4 days, vitamin K1, transfusion of two isogroup isorhesius blood bags, and oxygen therapy.

On 23 January 2018 (the sixth day of hospitalization), the patient died after repeated convulsions with bradypnea and then cardio-circulatory arrest.

The blood test result returned on 24 January 2018 and confirmed Lassa hemorrhagic fever by Reverse Transcriptase Polymerase Chain Reaction (RT-PCR) assay. A safe burial was organized. An investigation team has been set up to look for and follow up contact subjects under the supervision of the Epidemiological Surveillance Unit of the Borgou’s Departmental Health Office. Each contact subject was provided with a thermometer allowing him to take morning and evening body temperature and to communicate it to the focal point of the epidemiological surveillance for a total duration of 21 days as from the date of the contact with the case. All contact subjects gave their oral consent to participate in the follow-up. The data resulting from this follow-up were treated confidentially and anonymously. No associated cases were observed among contact subjects.

## Discussion and conclusions

This clinical case highlights the diagnostic difficulties of viral hemorrhagic fevers in general and Lassa fever in particular in the context of resource-limited countries. Several factors contribute to the delayed diagnosis. The lack of inadequacy of knowledge of the disease by healthcare workers plays a definite role in the delayed diagnosis [4]. The lack of specificity of clinical and paraclinical manifestations, especially at the beginning of the disease, makes it impossible to think about it very early [1, 5–8]. Clinical manifestations sometimes simulate a surgical emergency and diagnosis is only obtained post-operatively [9]. It can also take the form of clear liquid meningitis [10]. Diagnosis is facilitated only by the vigilance of healthcare personnel, especially in an epidemic context, and the persistence of symptoms despite good management of acute fever (malaria treatment, antibiotic therapy) [5, 11]. Our patient was not recognized as a suspected case of Lassa fever until 11 days after the onset of symptomatology when several signs consistent with the diagnosis appeared. These were aggravation of digestive bleeding, puffiness of the face, tonsillitis, and persistent fever despite triple antibiotic therapy. Indeed, bleeding is often perceived as one of the main signs suggestive of viral hemorrhagic fevers [12–14]. This sign attracts the attention of caregivers more often, whereas it is present in only 5 to 70% of cases and only appears at the third progressive stage of the disease [1, 15]. Hence the needs to regularly train all caregivers on the definition of Lassa fever cases. It is also necessary to inform them well about the notification circuit of suspicious cases and the measures to be taken at their level until the case enters the formal care circuit. Our patient’s diagnosis could have been facilitated by the notion of regular consumption of rat meat. But all indications are that the patient withheld information. Indeed, information on the stay on a farm and the consumption of rats was only given very late in the face of the worsening of its clinical condition. Reasons for withholding information are often the fear of being abandoned by caregivers or isolated. Isolation is generally poorly experienced and culturally poorly perceived by patients and their families. It is therefore necessary to develop strategies for rapid diagnosis of viral hemorrhagic fevers and to make them accessible at all levels of the health system. Indeed, in the Beninese context, all samples are sent to a single laboratory located in Cotonou, and the result is available at least 48 h later. However, rapid diagnosis, whether negative or positive, allows for adequate patient management, limits the risk of exposure of caregivers, and reassures both the patient and his parents. Regarding antiviral treatment, our patient was put on Ribavirin as soon as Lassa fever was suspected but he died after only 1 day of treatment. We received confirmation of the diagnosis after his death. In reality, antiviral treatment is of interest only when it is started early [13]. This confirms once again the need for early diagnosis of the disease.

In conclusion, effective control of Lassa fever in Benin will involve training healthcare workers on the signs of the disease, defining cases and organizing their management, which requires rapid confirmation of the diagnosis. It is therefore necessary to have the means for rapid detection of these diseases and to make them accessible at all levels of the health system. This strategy will be reinforced by information and education campaigns for the population on the means of transmission and prevention of the disease and especially on the need to go as quickly as possible to a health facility in case of fever.

## Abbreviations

ALT: Alanine aminotransferases
AST: Aspartate aminotransferases
HIV: Human immunodeficiency virus
IV: Intravenous
RT-PCR: Reverse Transcriptase – Polymerase Chain Reaction
